# Supplementary material for: Improving fishing ground estimation with weak supervision and meta-learning
Source: PLoS One. 2025 Apr 11;20(4):e0321116. doi: 10.1371/journal.pone.0321116 (PMC11991730; doi:10.1371/journal.pone.0321116)
Supplement: Attachment — Submitted filename: Response to Reviewers.pdf [file pone.0321116.s002.pdf]

Response to Reviewers

We sincerely thank the reviewers for carefully reading our manuscript and giving us constructive comments to improve our paper. We have made changes that reflect most of the suggestions we received from the reviewers. We have highlighted the changes in the manuscript.

The following is a point-by-point response to the reviewers' comments.

[Comment:1] *The introduction contains a lot of information but lacks a clear paragraph structure. Please divide the introduction into several paragraphs, each focusing on a specific topic, such as research background, limitations of existing methods, and contributions of this paper.*

[Response]

Thank you for pointing out the need for a clearer paragraph structure in the Introduction. We agree that a clearer paragraph structure would enhance the readability and organization of the introduction. In response, we have thoroughly revised the Introduction section to address your concerns. The updated structure is as follows:

- Paragraph 1: Research background and importance
- Paragraph 2: Existing studies and their limitations
- Paragraph 3: Pattern recognition in Fishing Ground Estimation and its limitations
- Paragraph 4: Proposed approach using weakly supervised pre-training
- Paragraph 5: Meta-learning to mitigate noisy labels

We believe that this revised structure will provide a more coherent and focused introduction to the research problem, existing methods, and the contributions of our work.

[Changes]

Lines 2-44

[Comment:2] *When describing the method, there is a lack of concise description of the specific details of keypoint detection.*

[Response]

Thank you for your insightful comment regarding the description of the keypoint detection method. We agree that the initial explanation lacked specificity. In response, we have revised

the "Keypoint Detection Model" section to provide a more detailed and concise description. Specifically, we now explain that we employed Lightweight OpenPose, a streamlined version of the original OpenPose architecture, for our fishing ground estimation. We clarified that while the original OpenPose outputs both keypoint heatmaps and part affinity fields to capture pairwise relationships between keypoints, our method focuses exclusively on the keypoint heatmaps. These heatmaps predict the likelihood of fishing grounds at each location on the SST map.

We also included details about the model's architecture, noting that it consists of multiple convolutional layers that progressively refine the heatmaps across stages. Additionally, we explained how both the input SST maps and the output heatmaps are resized to maintain spatial consistency, as illustrated in Fig. 7.

We believe these revisions address your concern by providing a clearer and more comprehensive description of the keypoint detection process in our method.

[Changes]

lines 233-243

[Comment:3] *The description of the data is not detailed enough, especially the process of data preprocessing and annotation generation.*

[Response]

Thank you for highlighting the need for more detailed descriptions of our data preprocessing and annotation generation. We have added two new sections, Pre-processing and Dataset Composition, to address this.

In Pre-processing, we now explain that we selected only single-line fishing vessels to focus on skipjack tuna. We applied an SST filter to exclude trajectory points outside the 19° C to 26° C range, which is typical for skipjack tuna habitats. For generating ground-truth heatmaps, we applied a Gaussian kernel with a standard deviation ($¥sigma$) of 7 to catch locations or averaged daily vessel positions. To prevent over-densification, we performed peak detection using an $8 ¥times 8$ maximum value filter.

In Dataset Composition, we detail the datasets used in our experiments:

- Pre-training: 2,087 days of trajectory data from 2012 to 2017.
- Fine-tuning and Confidence Network Training: 371 days of catch data from 2012 and 2015.
- Testing: 154 days of catch data from 2018.

Table 3 summarizes the number of images and instance counts across these phases.

We believe these additions provide the necessary detail to address your concern.

[Changes]
lines 289-309

[Comment:4] *The description of the experimental design and the division of the data set is not detailed enough. Please add detailed description of the experimental design, including the division of the data set (training set, validation set and test set), the selection of hyperparameters, evaluation indicators, etc.*
[Response]
Thank you for highlighting the need for more detail in our experimental design. We have added an Experimental Design section to provide detailed descriptions of the dataset division, hyperparameter selection, and evaluation metrics.

Dataset Division: We now explain how the data was split into training, validation, and test sets. Trajectory data from 2012–2017 was used for pre-training. Catch data from 2012–2015 was split into training and validation sets with a 0.2 validation ratio for fine-tuning and training the confidence network. Catch data from 2018 was reserved exclusively for testing.

Training and Hyperparameters: We describe our two-stage training process and list the hyperparameters used (Table4). Training epochs were determined based on validation metrics, and other hyperparameters were empirically tuned.

We believe these additions address your concerns.

[Changes]
Lines 309-325
