# Supplementary material for: Improving fishing ground estimation with weak supervision and meta-learning
Source: PLoS One. 2025 Apr 11;20(4):e0321116. doi: 10.1371/journal.pone.0321116 (PMC11991730; doi:10.1371/journal.pone.0321116)
Supplement: Attachment — Submitted filename: Response_to_Reviewers_auresp_2.pdf [file pone.0321116.s003.pdf]

Response to Reviewers

We sincerely thank the reviewers for carefully reading our manuscript and providing us with constructive comments to improve our paper. We have made changes that reflect most of the suggestions we received from the reviewers. We have highlighted the changes in the manuscript.
The following is a point-by-point response to the reviewers' comments.

[Comment:1]

The title emphasizes the methodology ("weakly supervised keypoint detection and meta-learning") but does not highlight the outcome or potential implications (e.g., enhanced fishing efficiency, improved ecological management). Therefore, I would suggest to shift the focus toward the practical impact of the research. For example: "Improving Fishing Ground Predictions with Weak Supervision and Meta-Learning".

[Response:1]

We appreciate the reviewer's suggestion regarding the title. We have revised it to "Improving Fishing Ground Estimation with Weak Supervision and Meta-Learning," as it maintains clarity while aligning with the terminology used in the main text. While "prediction" could also be appropriate, we chose "estimation" by analogy with Human Pose Estimation, which similarly involves inferring spatial patterns from data.

[Comment:2]

While the abstract mentions "effectiveness of pre-training and meta-learning," it does not provide quantitative outcomes or a concrete comparison to existing methods. Including key performance metrics (e.g., improvement in F1-score) would better highlight the study's contributions. Additionally, terms like "weakly supervised pre-training" and "meta-learning" may be unclear to readers unfamiliar with machine learning. A brief simplification or explanation of these terms would improve accessibility.

[Response:2]

We appreciate the reviewer's suggestion to enhance the clarity of the abstract. In response, we have included key performance metrics, specifically highlighting the 64% improvement in F1-score (from 0.055 to 0.09) compared to the baseline using only catch data. Additionally, we have simplified and briefly explained "weak supervision" and "meta-learning" to make the abstract more accessible to readers unfamiliar with machine learning.

[Comment:3]

In my opinion, while the introduction highlights the scarcity of annotated data as a motivation for weakly supervised learning, it lacks a broader context about how this approach compares to or complements other learning paradigms in similar domains (e.g., transfer learning, semi-supervised learning). Also, the focus on pattern recognition in sea surface temperature (SST) data could be enriched by drawing analogies to neural mechanisms involved in processing spatiotemporal patterns, such as those in the visual cortex. In this regards, authors might consider further discussing how the decision-making process in fishers parallels neural mechanisms in humans, such as how the prefrontal cortex integrates environmental cues (e.g., SST patterns) to make predictions and decisions [; ;].

[Response:3]

We appreciate the reviewer's suggestion to provide a broader context by discussing transfer learning, semi-supervised learning, and other related paradigms. In response, we have explicitly incorporated transfer learning into the discussion, clarifying how it compares to our weakly supervised learning approach. Specifically, we highlight that while transfer learning typically relies on large, general-purpose pre-training datasets, our setting involves weak and imprecise labels, making weakly supervised learning more suitable. The aspects of semi-supervised learning and noisy label learning were already covered in our discussion.
Regarding the analogy to neural mechanisms in human decision-making, we acknowledge that drawing such parallels could provide interesting insights. However, our focus is on modeling fishers' decision-making

heuristics rather than analyzing the specific cognitive or neural mechanisms underlying their judgments. While human decision-making processes may involve the neural integration of environmental cues (e.g., SST patterns), our study does not aim to explore these biological aspects. Instead, we focus on the computational modeling of how fishers interpret and utilize SST patterns based on their experience.

[Comment:4]

The reliance on fishing vessel trajectory and catch data limits the scope of generalizability. Additional sources, such as environmental factors (e.g., sea current velocity, chlorophyll levels), could enrich the dataset and enhance model accuracy.

[Response:4]

We appreciate the reviewer’s suggestion to incorporate additional environmental factors, such as sea current velocity and chlorophyll levels. While fishing vessel trajectory and catch data are central to the current study, we agree that incorporating environmental variables could enrich the dataset and potentially enhance model performance.
The current focus was to demonstrate the feasibility of our weakly supervised pre-training and meta-learning approach using available data. However, incorporating environmental factors is certainly a valuable direction for future work, and we intend to explore their impact on model performance in subsequent studies, as mentioned in the limitations section.

[Comment:5]

The F1-scores for the proposed method are significantly low, especially compared to traditional keypoint detection tasks. This discrepancy needs a deeper discussion of potential causes and strategies for improvement beyond the suggestions in the limitations section.

[Response:5]

We appreciate the reviewer’s observation regarding the relatively low F1 scores of our method compared to traditional keypoint detection tasks. This discrepancy stems from the intrinsic challenges of estimating fishing grounds, which are inherently more ambiguous than tasks like human pose estimation. Unlike human keypoints, which follow clear anatomical constraints, fishing grounds are influenced by complex, dynamic environmental factors and incomplete observational data. Additionally, the noise in trajectory-based supervision and the limitations of available catch data further impact performance.
To clarify these points, we have expanded the discussion on the factors contributing to lower F1 scores and potential strategies for improvement in the limitations section.

[Comment:6]

The geodesic distance threshold (200 km) for evaluating accuracy might be overly lenient. A stricter metric could provide better insights into the method's precision and applicability.

[Response:6]

We acknowledge that this criterion may be considered lenient. A stricter threshold could indeed provide more precise insights into the method's accuracy. However, in the context of our research, the primary objective is to demonstrate the relative improvement achieved by the proposed method, rather than focusing on the absolute threshold.

[Comment:7]

The paper highlights noisy and imprecise trajectory data influencing pre-training. The justification for using trajectory data as weak supervision requires further validation to strengthen the rationale for this approach.

[Response:7]

We appreciate the reviewer’s concern regarding the use of fishing vessel trajectory data as weak supervision. Our approach follows a widely used strategy in machine learning, where easily obtainable but noisy labels serve as substitutes for direct annotations, as seen in ¥cite{jia2021scaling}. Similar to how large-scale vision-

language models leverage noisy text-image pairs to scale representation learning, we use trajectory-based fishing activity inference as a proxy for direct fishing ground annotations. This is a practical solution given the difficulty of obtaining precise, large-scale ground-truth labels for fishing grounds. Additionally, prior works ¥cite{Guan2021, Armas2022, Adibi2019, Maina2016} have successfully employed vessel speed and movement patterns to infer fishing ground locations, further supporting our methodology. We have clarified this rationale in the related work section.

[Comment:8]

The use of meta-learning to mitigate noisy labels is innovative, but the paper lacks quantitative comparisons against simpler alternatives (e.g., data augmentation or filtering). Demonstrating the necessity and added value of meta-learning in this context would solidify the contribution.

[Response:8]

We appreciate the reviewer's suggestion to compare our approach with simpler alternatives, such as data augmentation or filtering. In our study, filtering was already incorporated as part of the labeling and pre-processing stages to ensure data consistency and reduce noise. Specifically, we applied an SST filter to exclude data points outside the typical temperature range for skipjack tuna and only selected single-line fishing vessels for analysis. Additionally, the labeling process itself helped mitigate label noise by distinguishing between different fishing ground categories.
Regarding data augmentation, techniques like flipping or rotation are often effective in vision-based tasks, but they are not appropriate in our setting due to spatial and environmental constraints. Applying such augmentations would introduce unrealistic distortions to the geographical and oceanographic context of the data, likely leading to degraded model performance. Therefore, it is evident that simple alternatives would result in lower performance compared to our proposed method.

[Comment:9]

While the authors compare their method to alternatives like "catch only" and "fine-tuning," the addition of comparisons to state-of-the-art methods for weakly supervised learning in similar domains could enhance the impact of the results.

[Response:9]

We appreciate the reviewer's suggestion to include comparisons with state-of-the-art methods in weakly supervised learning. Our study already includes references to relevant state-of-the-art approaches in this area, such as those presented by Jia et al. (2021), Joulin et al. (2015), Dehghani et al. (2017), and Zhang et al. (2021). These methods are represented in our experiments as the "w/o confidence" setting, where we performed weakly supervised pre-training using trajectory data without the inclusion of confidence values.

[Comment:10]

The choice of Lightweight OpenPose for keypoint detection is reasonable, but an ablation study exploring the impact of alternative architectures (e.g., High-Resolution Networks or DeepLab) could provide more depth to the findings.

[Response:10]

We appreciate the reviewer's suggestion regarding alternative architectures. However, the core contribution of our study lies in the weakly supervised pre-training and meta-learning framework rather than in the specific model architecture. Our method is designed to be adaptable to different backbone networks, and while exploring alternative architectures could yield marginal performance variations, we do not expect it to fundamentally change the effectiveness of our approach.
Given our focus on leveraging weak labels and meta-learning, we prioritized a lightweight and efficient architecture that balances performance and computational feasibility. Nonetheless, we acknowledge the potential benefits of testing alternative models and will consider this in future work, as mentioned in the limitations section.

[Comment:11]

The experimental focus on skipjack tuna limits the study's broader applicability. Expanding the analysis to include multiple species or regions could make the method more universally relevant.

[Response: 11]

We acknowledge the reviewer's concern regarding the study's focus on skipjack tuna. Our primary goal was to develop and validate the proposed method within a well-defined and ecologically significant use case. Skipjack tuna was chosen due to its commercial importance and the availability of relevant trajectory and catch data. While the current study does not explicitly evaluate multiple species or regions, the methodology itself is not species-specific and can be extended to other fisheries with appropriate data. We will consider expanding the analysis in future work to further demonstrate the generalizability of our approach.

[Comment:12]

Figures showing heatmaps and output predictions (e.g., Fig. 11) could benefit from enhanced clarity and consistent formatting. Overlapping visualizations could obscure key findings, so better differentiation between estimated and ground-truth points would help.

[Response: 12]

We appreciate the reviewer's suggestion regarding the clarity and formatting of the heatmap figures. In response, we have revised Fig. 11 by enhancing the differentiation between estimated and ground-truth points to make the comparisons more visually distinct. Additionally, we cropped the area to zoom in on key regions, improving the readability of fine-grained details.

[Comment:13]

The results using hinge loss indicate decreased precision in some cases. This requires a more thorough examination and explanation of why hinge loss might not align with the task's objectives.

[Response: 13]

We appreciate the reviewer's insightful observation regarding the impact of hinge loss on precision. In response, we have conducted a more thorough examination of why hinge loss may not fully align with our task objectives. We clarified that hinge loss is designed to mitigate under-detection by removing the penalty for over-detection, which increases the number of predictions. However, our analysis revealed that this can lead to the inclusion of unsuitable fishing locations, particularly when the model has already been pre-trained to generate more predictions. As a result, precision decreases without a meaningful increase in recall, particularly in the with confidence condition.
To address this, we have expanded our discussion in the change of loss function section to emphasize the trade-off between recall and precision in our specific task. While hinge loss is effective in preventing under-detection, it requires further optimization to prevent excessive over-detection.
